# Unmasking structural patterns in incidence matrices: an application to ecological data

**DOI:** 10.1098/rsif.2018.0747

**Published:** 2019-02-06

**Authors:** Bernat Bramon Mora, Giulio V. Dalla Riva, Daniel B. Stouffer

**Affiliations:** 1 Centre for Integrative Ecology, School of Biological Sciences, University of Canterbury, Christchurch, New Zealand; 2 School of Mathematics and Statistics, University of Canterbury, Christchurch, New Zealand

**Keywords:** null models, ecological networks, species assemblages, structural patterns, network motifs, nestedness

## Abstract

Null models have become a crucial tool for understanding structure within incidence matrices across multiple biological contexts. For example, they have been widely used for the study of ecological and biogeographic questions, testing hypotheses regarding patterns of community assembly, species co-occurrence and biodiversity. However, to our knowledge we remain without a general and flexible approach to study the mechanisms explaining such structures. Here, we provide a method for generating ‘correlation-informed’ null models, which combine the classic concept of null models and tools from community ecology, like joint statistical modelling. Generally, this model allows us to assess whether the information encoded within any given correlation matrix is predictive for explaining structural patterns observed within an incidence matrix. To demonstrate its utility, we apply our approach to two different case studies that represent examples of common scenarios encountered in community ecology. First, we use a phylogenetically informed null model to detect a strong evolutionary fingerprint within empirically observed food webs, reflecting key differences in the impact of shared evolutionary history when shaping the interactions of predators or prey. Second, we use multiple informed null models to identify which factors determine structural patterns of species assemblages, focusing in on the study of nestedness and the influence of site size, isolation, species range and species richness. In addition to offering a versatile way to study the mechanisms shaping the structure of any incidence matrix, including those describing ecological communities, our approach can also be adapted further to test even more sophisticated hypotheses.

## 1. Introduction

Null models are an integral part of modern ecology and biogeography and provide a crucial statistical tool to test hypotheses regarding phenomena such as community assembly [1–5]. The underlying idea behind the use of any null model is that comparisons of real data to randomly generated data can provide insights into how biological data are structured, as well as the potential mechanisms explaining such structure. Following this idea, a structural pattern found in an observed biological system is only regarded as statistically meaningful if it is not reproducible by a random model and is therefore unlikely to be found purely by chance.

In the case of ecological networks or species assemblages, null models are often based around sampling and shuffling species’ interactions or presence/absence data [6], respectively. Therefore, the specific null hypothesis that is being tested with such a null model is entirely defined by the underlying randomization strategy [2,7,8]. As such, standard practice is for the randomization strategy of any null model to be generated in a way that includes some biological information while intentionally excluding other information. The differences observed between the empirical data and the data generated by the null model are then assumed to be a direct consequence of the omission of such information.

This approach, however, has not been without some controversy, since the choice of an inadequate null model may lead to artefactual conclusions [3,9]. For example, using null models to identify the mechanisms underlying the structure of biological data can be ambiguous, because there is not always a single way of introducing specific information into a model; therefore, these hypothesized mechanisms can only be supported by some evidence rather than a definitive proof. Perhaps more importantly, the randomization strategy may neglect some factors that could be responsible for a particular structural pattern. At times, this omission is due to insufficient prior evidence to support the idea that some unforeseen factor is potentially an important driver. At others, it arises due to the apparent difficulty with which to include such information into the randomization strategy. For example, null models employed to community-scale data in ecology often ignore the fact that species are part of a hierarchically structured phylogeny [10], and thus, the idea that observed structural regularities may potentially be explained most parsimoniously as the outcome of a complex evolutionary process [11,12].

This present work is an attempt to overcome the aforementioned difficulties by combining the classic concept of a null model and the ideas underlying joint modelling in community ecology. Joint models are a set of statistical tools for integrating environmental predictors and species interactions into a common framework [13]. These tools have been very helpful for understanding species richness and co-occurrence in ecological communities [14,15], and we use them here to expand beyond the traditional null model approach. In particular, we present a correlation-informed null model that flexibly incorporates biologically relevant information as an ingredient for the null hypotheses as opposed to post-hoc tests of the influence of those factors on the structure of biological data or on null model comparisons [16,17]. For example, given a particular ecological community, a correlation-informed null model generates a random community that is informed by any given correlation matrix. This new approach therefore provides a methodological framework to assess the importance of any measurable species trait (e.g. phylogenetic relatedness, body size or species’ tolerance to environmental conditions), habitat properties (e.g. ecosystem type, geographical distance or altitude) or combinations of these, on the structural patterns observed within such community data.

In order to demonstrate the versatility and power of the method presented here, we revisit examples from the literature that are emblematic of common problems encountered across community ecology. First, we apply the method to test whether or not a null model accounting for species’ shared evolutionary history can reproduce the structural properties observed in empirical food webs. To do so, we use a phylogenetically informed null model, which allows us to evaluate whether or not the structure of empirical and simulated food webs appears non-random when accounting for potential conservation of interactions. Second, we analyse the factors that influence the structure of species assemblages, focusing in particular on the effect of non-independence between sample sites. Using different correlation-informed null models, we unmask the factors of one of the most used patterns in island biogeography studies. Though we have chosen to frame the methodology in an ecological context, note that the correlation-informed null model can be generalized to study the structure of any system that can be represented by association data and whose components can be related by an underlying correlation structure.

## 2. Material and methods

### 2.1. The null-model approach

#### 2.1.1. Uninformed null models

The structure of many systems is commonly described using an incidence matrix. This incidence matrix *A* describes the relationship between two given interacting sets {*i*} and {*j*}, where every element of the matrix *A*_*ij*_ is set to 1 when a relationship between *i* and *j* is present in the community, and 0 otherwise. For example, in ecology, a species assemblage can be conveyed by a matrix representing the presence/absence of different species across a set of sites, whereas an ecological network can similarly be represented by a matrix characterizing the presence/absence of interactions between two sets of species (e.g. predators and prey, plants and pollinators, or hosts and parasites). For the sake of simplicity, in the remainder of the methods, we will call any element *A*_*ij*_ = 1 a ‘link’ i←j even though in species assemblages this would not be an interaction in the standard ecological sense of the term.

With limited exceptions (e.g. [18] or [19]), the statistical significance of any structural pattern in an incidence matrix is conditioned to the chosen null hypothesis [8], which is generally described by an ensemble of randomized matrices. The vast majority of null models can follow either a probabilistic or a fixed algorithm to generate such randomized matrices [20]. The probabilistic approach samples the matrix elements based on the total number of links of both row and column elements [21,22], preserving approximates of their overall distributions. The fixed strategy, on the other hand, randomizes the possible links by either recursively swapping the existing ones (‘swap’ algorithm; [23]) or randomly creating them (‘fill’ algorithm; [24]), in such a way that they exactly match constraints imposed by row and column marginals [25].

The randomization strategy used here is based around the swap algorithm [23]—also referred to as fixed-fixed null model. That is, we use a Markov chain Monte Carlo switching algorithm to iteratively select existing links and swap them, provided that these swaps agree with the imposed constraints [26,27]. For instance, for the purpose of randomizing a matrix *A* while preserving both the degree of row and column elements, the algorithm would repeatedly select two existing links i←j and l←m at random, and transform them into i←m and l←j on the condition that they are not already present in the community. Importantly, the standard form of this randomization strategy swaps any pair of existing links with equal prior probability. That is, in every iteration of the randomization process, the selection criteria for the choice of the swapping links is uninformed, implying that any two links are equally likely to be shuffled as long as such shuffling agrees with the other imposed structural constraints.

Note that the swap algorithm does not establish a minimum number of iterations—also referred to as ‘swap trials’—needed in order to obtain fully randomized incidence matrices; this will depend on the size and structure of the incidence matrix being randomized. Miklós & Podani [28] recommend ensuring that the number of trials is such that the expected number of actual swaps is twice the number of 1’s in the incidence matrix. For the purpose of studying structural patterns in randomized incidence matrices, however, we would suggest making sure that an increase in the number of swap trials does not lead to any changes to the average representation of such structural patterns.

#### 2.1.2. Correlation-informed null models

**Figure 1.**
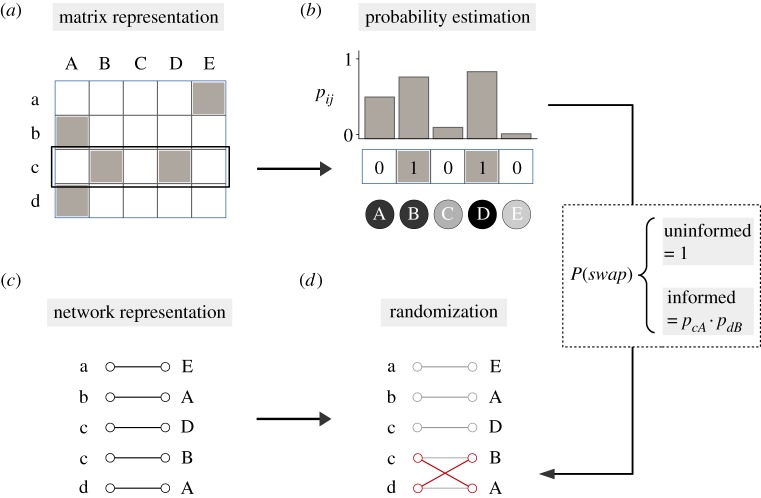
Graphical representation of the proposed randomization process. (*a*) The null model generates data by randomizing a given incidence matrix, where rows and columns represent two interacting sets {*x*} and {*Y*}, and the grey squares indicate an interaction between elements of the sets. (*b*) Based on some column attribute or trait (in this example the grey tone of the circles under the graph) and the empirical matrix, we can estimate the data-informed probability of encountering any of the possible interactions of the incidence matrix. (*c*,*d*) The randomization algorithm then repeatedly swaps two randomly selected links in the network representation of (*a*) according to the estimated probabilities. For example, if the algorithm selected links c←B and d←A, they would be swapped with probability $p=p_{cA}\cdot p_{dB}$ in the informed case. In the uninformed case, the swap would occur with probability *p* = 1. (Online version in colour.)

In contrast to the uninformed null model, we introduce a manner in which to ‘inform’ the swapping algorithm so that the probability of randomizing different links depends on underlying biological information—i.e. information on additional constraints or tendencies in the natural phenomena. To do so, we modify the randomization process in order to account for the information encoded within a specified correlation matrix. Specifically, we calculate estimates of the probability to observe any given link in an incidence matrix by means of a generalized linear mixed model [29–32]. Given a row *i* from an incidence matrix *A* of size *n* × *m* and a correlation matrix *V*_col_ of size *m* × *m* relating the corresponding *m* columns, the probability of observing a link between row *i* and column *j* can be estimated by fitting the observed links *A*_*ij*_ to the following logistic regression:

2.1 $$\text{logit}\;(p_{ij})=\alpha_{i}+b_{ij},$$

where *α*_*i*_ is a constant intercept and *b*_*ij*_ is a Gaussian distributed random effect with mean 0 accounting for the correlation matrix (figure 1). The covariance matrix of *b*_*ij*_ is $\sigma_{i}^{2}V_{\mathrm{col}}$, which represents an estimated scalar multiplied by the *m* × *m* correlation matrix. Therefore, the estimation of the scalar *σ*_*i*_ roughly reveals how well the observed links can be predicted by the correlation matrix *V*_col_ [29]. As this regression is performed for every row *i* of the incidence matrix *A*, the sample size over which the parameters are inferred is exactly the number of column elements *m* contained in each row. Note that this same estimation can be performed for a correlation matrix *V*_row_ that relates the *n* rows by instead fitting the model to the transpose of the incidence matrix.

The estimated probabilities *p*_*ij*_ provide then a way of weighting the randomization process based on the correlation matrix. That is, we can introduce a bias in the null model so that the swap algorithm transforms two randomly selected links i←j and l←m into i←m and l←j according to the joint probability *p*_*im*_ × *p*_*lj*_, again provided that this change agrees with any other potential constraints imposed on the model (figure 1). This bias to the null model defines the correlation-informed null model. Note that the estimation of the probabilities *p*_*ij*_ is done prior to the randomization process; therefore, every step of the swap algorithm is informed relative to the original incidence matrix. Also, it is worth pointing out that the chosen joint probability assumes independence of interactions, and more sophisticated approaches could also be taken into consideration when combining the estimated probabilities.

Importantly, the correlation matrix used to fit the incidence matrix can (i) either provide valuable information to explain the observed links or (ii) appear completely uninformative to them. In the former case, the estimated probabilities will present a heterogeneous pattern whereas in the latter case they will tend to show a uniform distribution. Moreover, an informative correlation matrix does not imply a more predictive null model since the information provided might be irrelevant to explain the particular pattern that is ultimately being tested.

#### 2.1.3. Misinformed null models

Following the definition of the correlation-informed null model, we can also define a misinformed null model, where the randomization process is itself informed by randomized correlation matrices. That is, given an incidence matrix *A* and correlation matrix *V*_col_, we generate every random network $A^{*}$ of a misinformed null model as follows: we first randomize *V*_col_, symmetrically permuting the row and column identities; we next estimate the probabilities *p*_*ij*_ using the randomized matrix V*_col_; and we finally use *p*_*ij*_ to ‘inform’ the swapping algorithm as described for the correlation-informed null model. A misinformed null model is necessary because it serves as a control model for the correlation-informed counterpart. This is because it allows us to test that a null model informed with the ‘wrong’ correlation structure—which is a form of overfitting—does not lead to artefactual conclusions. We expand on this below in the section ‘Model testing’ and in the electronic supplementary material, Methods.

#### 2.1.4. Correlation structures

Given an incidence matrix *A*_*n*×*m*_, the correlation matrix *V*_col_ = *V*_*m*×*m*_ (or *V*_row_ = *V*_*n*×*n*_) defines the relationships between the *m* column elements (or *n* row elements) of *A*. Every element *v*_*ij*_ = *v*_*ji*_ of this symmetric, positive semi-definite matrix characterizes the similarity between two columns (or rows) *i* and *j*. There are an infinite number of matrices that can be proposed as a correlation structure *V*_*m*×*m*_. For example, the most basic one would be a matrix such that every element *v*_*ij*_ is equal to 1, representing the case in which there are no differences across the *m* column elements. This basic case is important because such a correlation structure is not informative to the swap algorithm, and it produces a null model that behaves exactly as its uninformed counterpart. Alternatively, the *m* column elements could instead belong to different groups, and one could use these groups to define a correlation structure *V*_*m*×*m*_ such that *v*_*ij*_ = 1 if *i* and *j* belong to the same group, and 0 otherwise. This correlation structure would then inform the null model so that the randomization process is biased following such grouping.

Similar to the groups, one can generate a suitable correlation structure given any set of continuous values (or ‘traits’) that describe the *m* columns. Such a correlation structure can then take multiple forms, from a direct measure of similarity of these set of traits to other more sophisticated forms such as exponential or Gaussian structures. For instance, we could generate an exponential correlation structure *V*_*m*×*m*_ for a given set of column traits {*x*_*m*_} using

2.2 $$V_{\mathrm{col}}=(1-N)\exp\left(-\frac{D}{\max D}\right),$$

where *D* characterizes the distance matrix across all traits such that *d*_*ij*_ is the Euclidean distance between any two column traits *x*_*i*_ and *x*_*j*_, and *N* is a matrix such that every element in the diagonal *n*_*ii*_ = 0 and any other element *n*_*ij*_ = *η*. The factor *η* characterizes the ‘nugget effect’ for this correlation matrix, which is used as a way to avoid perfectly correlated off-diagonal elements. The computation of many well-known correlation structures can be done using functions within the R package *nlme* [33]; note, however, that the appropriateness of each will depend on the precise question being studied.

#### 2.1.5. Quantifying over- and underrepresentation

To test whether or not any structural pattern observed in an empirical incidence matrix is significantly non-random compared to the data generated by a null model, we use the pattern’s *z*-score. To understand this comparison, let us define the measure of an arbitrary structural pattern *k* = *k*(*A*) of an adjacency matrix *A*. This property could characterize simple aspects of the adjacency matrix such as the total number of links or other more complex metrics of the way in which the links are distributed within the matrix. Following this, such a structural pattern could also be measured in an ensemble of randomized matrices {A*} generated by a given null model, defining a null distribution of measures $\left\{k^{*}\right\}$. The pattern’s *z*-score can then be defined as

2.3 $$z=\frac{k-\langle \{k^{*}\}\rangle }{\sigma_{\left\{k^{*}\right\}}},$$

where $\left\langle \{k^{*}\}\right\rangle$ is the average measure of the structural pattern in the random ensemble and $\sigma_{\left\{k^{*}\right\}}$ is the corresponding standard deviation. A positive *z* indicates that the observed pattern is overrepresented in the empirical matrix, and significantly so for values greater than 1.96. Likewise, a negative *z* indicates that the pattern is underrepresented, and the threshold for significance is − 1.96.

### 2.2. Applications to ecological data

#### 2.2.1. Food webs and network motifs

The first emblematic example that we revisit from the literature is the study of the evolutionary history behind food-web structure. In particular, we studied how well species’ evolutionary relationships can explain observed patterns of interaction in food webs. To do so, we analysed 10 empirical food webs from small streams of the Taieri River in New Zealand comprising fish, macroinvertebrates and algae [34]. They are taxonomically highly resolved food webs—taxonomically or trophically related species were always considered independently—and range in size from 78 to 113 species. These food webs are from habitats that present many similarities (i.e. all sites were from grassland catchments and included at least one pool and one riffle) but still differ in fundamental ways (including but not limited to different size, altitude, stream depth and land-use).

For each of the 10 food webs, we focused on the analysis of the so-called food-web motifs—connected sub-graphs representing the different patterns of interactions between a subset of species [35]. The frequency of appearance of each of these subgraphs within a network defines a structural property that has proven to be a very powerful network metric to understand food-web structure [36]. When compared to a null hypothesis, this network metric has been shown to be very non-random, presenting consistent patterns of over- and under-representation [35,37–40]. We specifically focused on the study of the frequency of appearance of three-species food-web motifs, which have already been shown to be non-randomly represented in the dataset used here [35]. To do so, we used the tools provided by the Python module ‘pymfinder’ [36].

#### 2.2.2. Species assemblages and nestedness

As a second example, we analysed different factors that have been shown to influence the structure of species assemblages. Specifically, we explored how well possible spatial autocorrelations or area similarity between sample sites as well as island species richness and species range similarity can explain the structural patterns observed in these communities. To do so, we used the floristic database published by Marx *et al.* [41], which reports the distribution of 366 species of vascular plants across 80 islands from the San Juan archipelago [42]. The data were compiled between 2005 and 2010 and restricted to the smaller islands of the archipelago (less than 25 ha). This database also provides information on the size and geographical centroid of the islands.

In this case, we focused on the study of nestedness [43], a common measure of assemblage structure. A species-sites assemblage is said to be nested when sites with fewer species contain a subset of the species present in more abundant sites. Although there are multiple algorithms that define a measure for nestedness [44], we used the nestedness calculator NODF [45], which returns a value close to 100 when the community is highly nested and close to 0 otherwise.

#### 2.2.3. Model testing

To validate the models before analysing the empirical data, we benchmark tested them using artificially generated structured and random data. We decided to use two tests that mimicked the two empirical datasets chosen to introduce the method. In particular, we first generated artificial food webs and species assemblages and informative correlation matrices for their components (electronic supplementary material, Methods). Then, we studied the motif representation and nested patterns found in the food webs and species assemblages, respectively, comparing the performance of the uninformed, correlation-informed, and misinformed null models (electronic supplementary material, Results). As expected, we found the uninformed and misinformed null models to showcase very similar performance—showing similar patterns of over- and under-representation—while the correlation-informed null model was instead able to shed light on the structure of the generated data (electronic supplementary material, figure S3). This is important because it implies that correlation structures encoding information regarding the process in which the data are generated are informative to the null model, but other unrelated correlation structures do not affect the model’s performance. Finally, we performed the same tests using random data, where all the models showed the same over- and underrepresentation of the structural patterns (electronic supplementary material, figure S3).

## 3. Results

### 3.1. Application to food webs

**Figure 2.**
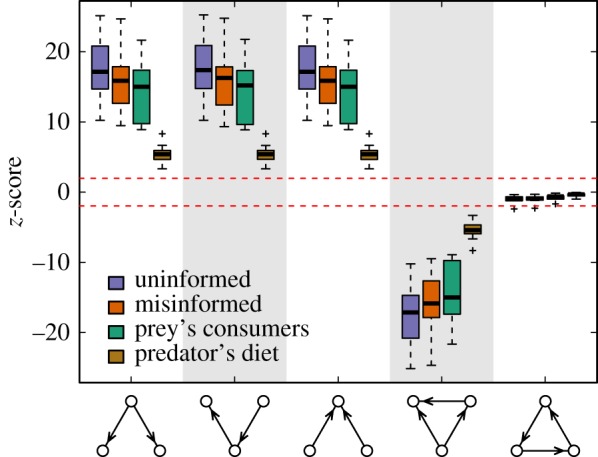
The effect of the phylogenetic relationships between species on the motif representation within a set of food webs. For all motifs, the arrow indicates the transfer of energy from prey to predators. The boxes contain the *z*-scores for each motif according to the different null models. The boxes group all food webs, extending from the lower to upper quartile values of the data, with a line at the median. The colour of the boxes indicates the null model used: an uninformed null model (uninformed), a misinformed null model (misinformed), a null model accounting for the phylogenetic relationships in preys’ consumers (prey’s consumers) and a null model accounting for the phylogenetic relationships in predators’ diets (predator’s diet). The dashed red line indicates the thresholds for significance *z* ≤ −1.96 and *z* ≥ 1.96. (Online version in colour.)

For each of the 10 empirical food webs, we first analysed the three-species motif representation using the uninformed null model. We generated data with this model by using fixed-fixed algorithm, shuffling species’ interactions while conserving each species’ number of prey and predators, and the distribution of single, double and cannibal links [27,35]. The reason for these constraints is that this type of randomization preserves the total numbers of prey and predators of all species and the two-species motif structures; therefore, it ensures that the over- or underrepresentation of a motif of size three is not due to the over- or underrepresentation of a particular sub-pattern [27,37,46]. We found that three different motif structures were significantly overrepresented in all 10 networks (figure 2): the motifs describing a simple food chain, exploitation competition, and apparent competition. We likewise found that the motifs representing omnivory and a three-species trophic loop were consistently underrepresented in every food web.

We then performed the same analysis using the phylogenetically informed null model. To do so, we first estimated phylogenies for the different species forming the 10 food webs under study (electronic supplementary material, Methods) and generated the corresponding phylogenetic covariance matrices using the function ‘vcv’ from the R package *APE* [47]. Then, we weighted the randomization strategy used in the uninformed case to account for the information encoded within the estimated phylogenies. To achieve this, we calculated the interaction probabilities of the food webs through equation (2.1), considering the phylogenetic covariance matrices as correlation matrices. These probabilities can be estimated following two different perspectives: the predator’s diet and the prey’s consumers. Given any interaction i←j, the former describes the probability of the predator *i* consuming *j* given the phylogenetic relationships between the prey species, whereas the latter represents the probability of the prey *j* being consumed by *i* given the phylogenetic relationships between the predator species.

With these two phylogenetically informed null models, we found the same pattern of over- and underrepresentation as that observed when using the uninformed null model (figure 2). In this case, however, the phylogeny appears to be particularly informative for determining food-web structure since data generated by the null model is much better at reproducing the empirical motif representation. Moreover, the results present key differences between the null model accounting for the phylogenetic relationships of predators’ diets and the one accounting for the phylogenetic relationships of preys’ consumers. Specifically, the motif profile is best preserved when we considered the predator’s diet perspective but is significantly less informative when the prey’s consumers perspective is adopted (figure 2). Importantly, the observed differences between the two informed null models were true even when controlling for the degree of overlap between the empirical food webs and their randomized counterparts (electronic supplementary material, Methods and Results). That is, such differences were not due to the number of shared links between the empirical and random structures but instead arose from the intrinsic properties of the adopted null hypotheses (electronic supplementary material, Results).

### 3.2. Application to species assemblages

**Figure 3.**
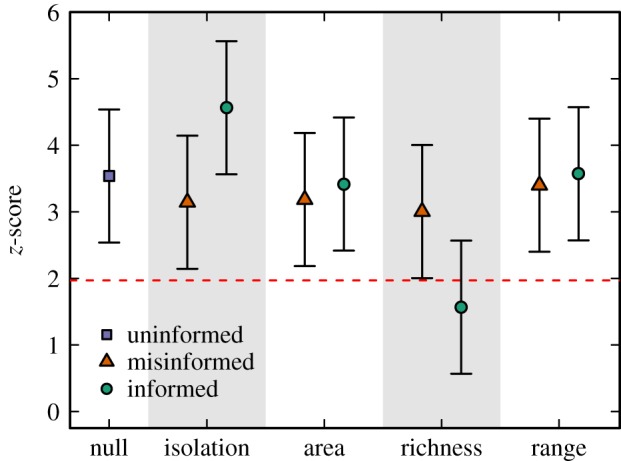
The effect of spatial autocorrelation, island area, island species richness and species range similarity on measures of community structure. We show the *z*-scores for the nestedness pattern in the distribution of vascular plants across islands from the San Juan archipelago. All plots show the results obtained using an uninformed null model (null), an isolation-informed null model (isolation), an area-informed null model (area), the richness-informed null model (richness) and the range-informed null model (range). The horizontal dotted line indicates the threshold for significance *z* ≥ 1.96. (Online version in colour.)

For the species assemblage data, we first analysed the nestedness pattern using the uninformed null model. We again followed the fixed-fixed algorithm, which is one of the most widely used approaches in biogeographic studies whereby the incidence matrix is randomized fixing both the number of species per site and the relative frequency of appearance of each species [28,48–50]. We observed that this species assemblage is more nested than expected by chance, presenting a significantly high *z*-score (figure 3).

Then, we used different informed null models to quantify the influence of the island isolation, island area, island species richness, and species range on the structure of this community. To do so, we first computed separate correlation structures for each of these factors. In particular, we assumed an exponential correlation by means of equation (2.2), which is a widely used approach to account for spatial autocorrelation in biogeographic studies [51]. For these particular examples, we used a nugget effect *η* = 0.01 to generate the correlation structure. Following this, we weighted the uninformed randomization process to account for the different correlation matrices using equation (2.1), as described in the Methods section.

The isolation-informed and area-informed null models, on one hand, showed the species assemblage to be significantly nested, presenting the same overall conclusion as the uninformed null model. That is, spatial autocorrelation and size similarity between islands in this database is not a significant predictor of the observed nested pattern (figure 3). On the other hand, the results obtained using the richness-informed and range-informed null models showed that while the difference in the relative frequency of appearance of each species is not a significant predictor of the observed nested pattern, the difference in the number of species per site is (figure 3). That is, the random matrices generated by the null model informed using the species range appeared significantly less nested than the empirical matrix; however, the random matrices generated by the null model informed using the island species richness appeared as nested as the empirical matrix (figure 3). In all cases, the results were also compared to the ones produced by misinformed null models, finding no apparent differences with the uninformed counterpart for this pattern.

## 4. Discussion

An extensive literature has been published about null models in ecology and biogeography [1,5,9], including models accounting for within-species spatial patterns [52,53]. Inspired by this work, we present here a general and flexible approach to study the mechanisms explaining the structure of biological communities. In particular, we combine the classic concept of a null model and the ideas underlying joint modelling to define a correlation-informed null model. This model allows us to assess how informative the information encoded within any given correlation matrix is for explaining the structural patterns observed within any incidence matrix. Using this approach, we focused on the study of the biological mechanisms shaping the structure of ecological networks and species assemblages. Specifically, we found (i) a strong phylogenetic component underlying food-web motifs and (ii) a nested pattern in species assemblages that seems to be predominantly explained by island species richness.

In the first application of the correlation-informed null model, we studied the phylogenetic signal behind species’ interactions. This idea was based on the long-held assumption and frequent observation that these interactions are evolutionarily conserved [11,12,54]. In particular, we compared uninformed, misinformed and phylogenetically informed null models to study the motif representation of empirical food webs. This comparison showed that the network’s motif profile is largely preserved in data generated by a null model accounting for the phylogenetic relationships in predators’ diets. By contrast, we found that this model is significantly less informative when the analogous prey’s consumers perspective is adopted. First, this observation showcases how biological mechanisms can be untangled using our approach. In particular, it supports the idea of a stronger phylogenetic signal in prey range for predators than in predator range for prey [55] as well as a prey-selection mechanism shaping the structure of food webs [35]. Importantly, although the effect of the phylogenetic information reveals itself as crucial to explain who interacts with whom in a food web, our results also highlight the fact that this is clearly insufficient to fully predict motif representation in prey–predator relationships.

In the second application of the correlation-informed null model, we analysed the patterns observed in species distributions across different habitats. We focused on the study of nestedness, which is a common measure employed in biogeographic studies. Nestedness has been associated with habitat variables such as area [16,56,57], isolation [16,58] or land quality [59]. Somewhat surprisingly, we found that neither isolation nor area differences between islands appear to provide particularly relevant information to explain the nested pattern observed in the distribution of vascular plants across islands from the San Juan archipelago. One potential explanation for this lack of predictive power could be the fact that the biggest islands of the archipelago were excluded from the analysis [41]. This notwithstanding, we found that island species richness can instead explain the nested pattern. This observation is important because it suggests that nestedness is perhaps little more than an artefact of island species richness that becomes tautological when one controls for it. Moreover, this observation is in contrast to the results found when using the range-inform the null models, which show that species range is instead a poor predictor of the nested pattern observed in the species assemblage.

As a key step moving forward, it could be worth adapting the strategies presented in this work to inform other models from neutral theory of island biogeography, expanding the framework to new randomization strategies. In addition, the scenarios presented here provide only an introduction of the possible applications for any such correlation-informed null model. For example, one could also focus on the structure of ecological bipartite networks (e.g. plant–pollinator, host–parasitoid, seed-dispersal, etc.) and the drivers determining observed non-random patterns (e.g. modularity, uniqueness, centrality, etc.). We could evaluate whether or not there is a dominant trait from a particular group shaping the interactions of those networks—e.g. nectar depths of plants or proboscis length of pollinators in plant-pollinator networks [60] and seed or beak size in seed-dispersal networks [61]. Alternatively, we could examine the structural differences observed between different network types and assess which are the factors explaining such differences—e.g. comparing pollination and herbivory network architectures accounting for the evolutionary relationships of their constituents [62].

Here, we have sought to showcase some of the basic applications of the correlation-informed null model, but there are many other questions that could be addressed using the same approach. For instance, the examples presented here have only considered the effect of a single correlation matrix; however, one could take into account higher-order correlations or simultaneously consider multiple correlation matrices to inform the same null model. Indeed, we could consider multiple random effects in equation (2.1) or combine the probabilities generated using multiple correlation matrices independently [29]. In an ecological context, this informed null model could study species assemblages by combining different habitat properties (e.g. soil characteristics, vegetation type, etc.) with multiple species traits (e.g. body size, phylogenetic relationships, etc.) into a generalized island biogeography study. Consequently, our correlation-informed null model offers a versatile way to study the mechanisms shaping the structure within biological data that can easily be adapted further to test even more sophisticated hypotheses. Perhaps more importantly, there are multiple systems and structural patterns outside the ecological realm for which a correlation-informed null model could be useful. Indeed, our model only requires a system whose structure can be represented as an incidence matrix. Therefore, similar analyses could be performed for systems such as protein–protein interaction networks, neuronal networks or transcriptional regulation networks, among many others.

## Supplementary Material

Supplementary Methods and Results

## Data accessibility

The method presented here is implemented as an R package [63] named *resoldre* and is available online at https://github.com/bernibra/RESOLDRE. The key components of *resoldre* call functions from the Rcpp [64] and RcppArmadillo [65] packages in order to speed up its use. The main function of this package is ‘randomize’, which can perform different informed randomizations of a given incidence matrix based on a specified correlation matrix relating its rows or columns. The data and a description of data files are also included in the same repository.

## References

[1] Gotelli NJ 2000 Null model analysis of species co-occurrence patterns. Ecology 81, 2606–2621. (10.1890/0012-9658(2000)081[2606:NMAOSC]2.0.CO;2)

[2] Gotelli NJ, Entsminger GL 2001 Swap and fill algorithms in null model analysis: rethinking the knight’s tour. Oecologia 129, 281–291. (10.1007/s004420100717) 28547607

[3] Manly BF 2006 Randomization, bootstrap and Monte Carlo methods in biology, vol. 70 Boca Raton, FL: CRC Press.

[4] Gotelli NJ, Ulrich W 2010 The empirical Bayes approach as a tool to identify non-random species associations. Oecologia 162, 463–477. (10.1007/s00442-009-1474-y) 19826839

[5] Miller ET, Farine DR, Trisos CH 2016 Phylogenetic community structure metrics and null models: a review with new methods and software. Ecography 40, 461–477. (10.1111/ecog.02070)

[6] Gotelli NJ 2001 Research frontiers in null model analysis. Glob. Ecol. Biogeogr. 10, 337–343. (10.1046/j.1466-822X.2001.00249.x)

[7] Ulrich W, Gotelli NJ 2013 Pattern detection in null model analysis. Oikos 122, 2–18. (10.1111/more.2013.122.issue-1)

[8] Rohr RP, Saavedra S, Bascompte J 2014 On the structural stability of mutualistic systems. Science 345, 1253497 (10.1126/science.1253497) 25061214

[9] Gotelli NJ, Graves GR 1996 Null models in ecology. Washington, DC: Smithsonian Institution Press.

[10] Cavender-Bares J, Kitajima K, Bazzaz FA 2004 Multiple trait associations in relation to habitat differentiation among 17 Floridian oak species. Ecol. Monogr. 74, 635–662. (10.1890/03-4007)

[11] Bersier L-F, Kehrli P 2008 The signature of phylogenetic constraints on food-web structure. Ecol. Complex. 5, 132–139. (10.1016/j.ecocom.2007.06.013)

[12] Gómez JM, Verdú M, Perfectti F 2010 Ecological interactions are evolutionarily conserved across the entire tree of life. Nature 465, 918–921. (10.1038/nature09113) 20520609

[13] Warton DI, Blanchet FG, O’Hara RB, Ovaskainen O, Taskinen S, Walker SC, Hui FK 2015 So many variables: joint modeling in community ecology. Trends Ecol. Evol. 30, 766–779. (10.1016/j.tree.2015.09.007) 26519235

[14] Pollock LJ, Tingley R, Morris WK, Golding N, O’Hara RB, Parris KM, Vesk PA, McCarthy MA 2014 Understanding co-occurrence by modelling species simultaneously with a joint species distribution model (JSDM). Methods Ecol. Evol. 5, 397–406. (10.1111/2041-210X.12180)

[15] Ovaskainen O, Hottola J, Siitonen J 2010 Modeling species co-occurrence by multivariate logistic regression generates new hypotheses on fungal interactions. Ecology 91, 2514–2521. (10.1890/10-0173.1) 20957941

[16] Wang Y, Bao Y, Yu M, Xu G, Ding P 2010 Nestedness for different reasons: the distributions of birds, lizards and small mammals on islands of an inundated lake. Divers. Distrib. 16, 862–873. (10.1111/j.1472-4642.2010.00682.x)

[17] Matthews TJ, Cottee-Jones HEW, Whittaker RJ 2015 Quantifying and interpreting nestedness in habitat islands: a synthetic analysis of multiple datasets. Divers. Distrib. 21, 392–404. (10.1111/ddi.2015.21.issue-4)

[18] Paine RT 1988 Food webs: road maps of interactions or grist for theoretical development? Ecology 69, 1648–1654. (10.2307/1941141)

[19] Dunne JA, Williams RJ, Martinez ND 2002 Network structure and biodiversity loss in food webs: robustness increases with connectance. Ecol. Lett. 5, 558–567. (10.1046/j.1461-0248.2002.00354.x)

[20] Fortuna MA, Stouffer DB, Olesen JM, Jordano P, Mouillot D, Krasnov BR, Poulin R, Bascompte J 2010 Nestedness versus modularity in ecological networks: two sides of the same coin? J. Anim. Ecol. 79, 811–817. (10.1111/j.1365-2656.2010.01688.x) 20374411

[21] Cook RR, Quinn JF 1998 An evaluation of randomization models for nested species subsets analysis. Oecologia 113, 584–592. (10.1007/s004420050412) 28308039

[22] Bascompte J, Jordano P, Melián CJ, Olesen JM 2003 The nested assembly of plant–animal mutualistic networks. Proc. Natl Acad. Sci. USA 100, 9383–9387. (10.1073/pnas.1633576100) 12881488 PMC170927

[23] Connor EF, Simberloff D 1979 The assembly of species communities: chance or competition? Ecology 60, 1132–1140. (10.2307/1936961)

[24] Sanderson JG, Moulton MP, Selfridge RG 1998 Null matrices and the analysis of species co-occurrences. Oecologia 116, 275–283. (10.1007/s004420050589) 28308537

[25] Gotelli NJ, Entsminger GL 2003 Swap algorithms in null model analysis. Ecology 84, 532–535. (10.1890/0012-9658(2003)084[0532:SAINMA]2.0.CO;2) 28547607

[26] Milo R, Kashtan N, Itzkovitz S, Newman ME, Alon U 2003 On the uniform generation of random graphs with prescribed degree sequences. (http://arxiv.org/abs/cond-mat/0312028).

[27] Itzkovitz S, Milo R, Kashtan N, Newman M, Alon U 2004 Reply to “comment on ‘subgraphs in random networks’ ”. Phys. Rev. E 70, 058102 (10.1103/PhysRevE.70.058102) 14525069

[28] Miklós I, Podani J 2004 Randomization of presence–absence matrices: comments and new algorithms. Ecology 85, 86–92. (10.1890/03-0101)

[29] Ives AR, Helmus MR 2011 Generalized linear mixed models for phylogenetic analyses of community structure. Ecol. Monogr. 81, 511–525. (10.1890/10-1264.1)

[30] Rafferty NE, Ives AR 2013 Phylogenetic trait-based analyses of ecological networks. Ecology 94, 2321–2333. (10.1890/12-1948.1) 24358717 PMC3874136

[31] Pearse WD, Purvis A, Cavender-Bares J, Helmus MR 2014 Metrics and models of community phylogenetics. In *Modern phylogenetic comparative methods and their application in evolutionary biology* (ed. L Zsolt Garamszegi), pp. 451–464. Berlin, Germany: Springer.

[32] Pearse WD, Cadotte MW, Cavender-Bares J, Ives AR, Tucker CM, Walker SC, Helmus MR 2015 pez: phylogenetics for the environmental sciences. Bioinformatics 31, 2888–2890. (10.1093/bioinformatics/btv277) 25948716

[33] Pinheiro J, Bates D, DebRoy S, Sarkar D, 2014 nlme: linear and nonlinear mixed effects models. See http://CRAN.R-project.org/package=nlme. R package version 3.1-117.

[34] Townsend CR, Thompson RM, McIntosh AR, Kilroy C, Edwards E, Scarsbrook MR 1998 Disturbance, resource supply, and food-web architecture in streams. Ecol. Lett. 1, 200–209. (10.1046/j.1461-0248.1998.00039.x)

[35] Stouffer DB, Camacho J, Jiang W, Amaral LAN 2007 Evidence for the existence of a robust pattern of prey selection in food webs. Proc. R. Soc. B 274, 1931–1940. (10.1098/rspb.2007.0571) PMC2275185 17567558

[36] Bramon Mora B, Cirtwill AR, Stouffer DB 2018 pymfinder: a tool for the motif analysis of binary and quantitative complex networks. *bioRxiv*, p. 364703.

[37] Milo R, Shen-Orr S, Itzkovitz S, Kashtan N, Chklovskii D, Alon U 2002 Network motifs: simple building blocks of complex networks. Science 298, 824–827. (10.1126/science.298.5594.824) 12399590

[38] Stouffer DB, Bascompte J 2010 Understanding food-web persistence from local to global scales. Ecol. Lett. 13, 154–161. (10.1111/ele.2010.13.issue-2) 19968697

[39] Baker NJ, Kaartinen R, Roslin T, Stouffer DB 2015 Species’ roles in food webs show fidelity across a highly variable oak forest. Ecography 38, 130–139. (10.1111/ecog.2015.v38.i2)

[40] Trøjelsgaard K, Olesen JM 2016 Ecological networks in motion: micro- and macroscopic variability across scales. Funct. Ecol. 30, 1926–1935. (10.1111/fec.2016.30.issue-12)

[41] Marx HE, Giblin DE, Dunwiddie PW, Tank DC 2015 Deconstructing Darwin’s naturalization conundrum in the San Juan Islands using community phylogenetics and functional traits. Divers. Distrib. 22, 1–14. (10.1111/ddi.12401)

[42] Marx HE, Giblin DE, Dunwiddie PW, Tank DC 2015 Data from: Deconstructing Darwin’s naturalization conundrum in the San Juan Islands using community phylogenetics and functional traits. See 10.5061/dryad.m88g7.

[43] Patterson BD, Atmar W 1986 Nested subsets and the structure of insular mammalian faunas and archipelagos. Biol. J. Linnean Soc. 28, 65–82. (10.1111/bij.1986.28.issue-1-2)

[44] Ulrich W, Almeida-Neto M, Gotelli NJ 2009 A consumer’s guide to nestedness analysis. Oikos 118, 3–17. (10.1111/oik.2009.118.issue-1)

[45] Rodríguez-Gironés MA, Santamaría L 2006 A new algorithm to calculate the nestedness temperature of presence–absence matrices. J. Biogeogr. 33, 924–935. (10.1111/jbi.2006.33.issue-5)

[46] Artzy-Randrup Y, Fleishman SJ, Ben-Tal N, Stone L 2004 Comment on ‘network motifs: simple building blocks of complex networks’ and ‘superfamilies of evolved and designed networks’. Science 305, 1107–1107 (10.1126/science.1099334) 15326338

[47] Paradis E, Claude J, Strimmer K 2004 APE: analyses of phylogenetics and evolution in R language. Bioinformatics 20, 289–290. (10.1093/bioinformatics/btg412) 14734327

[48] Ulrich W, Gotelli NJ 2007 Null model analysis of species nestedness patterns. Ecology 88, 1824–1831. (10.1890/06-1208.1) 17645028

[49] Almeida-Neto M, Ulrich W 2011 A straightforward computational approach for measuring nestedness using quantitative matrices. Environ. Modell. Softw. 26, 173–178. (10.1016/j.envsoft.2010.08.003)

[50] Strona G, Fattorini S 2014 On the methods to assess significance in nestedness analyses. Theory Biosci. 133, 179–186. (10.1007/s12064-014-0203-1) 24974139

[51] Dormann CF *et al.* 2007 Methods to account for spatial autocorrelation in the analysis of species distributional data: a review. Ecography 30, 609–628. (10.1111/j.2007.0906-7590.05171.x)

[52] Roxburgh SH, Chesson P 1998 A new method for detecting species associations with spatially autocorrelated data. Ecology 79, 2180–2192. (10.1890/0012-9658(1998)079[2180:ANMFDS]2.0.CO;2)

[53] Roxburgh SH, Matsuki M 1999 The statistical validation of null models used in spatial association analyses. Oikos 85, 68–78. (10.2307/3546792)

[54] Rezende EL, Albert EM, Fortuna MA, Bascompte J 2009 Compartments in a marine food web associated with phylogeny, body mass, and habitat structure. Ecol. Lett. 12, 779–788. (10.1111/ele.2009.12.issue-8) 19490028

[55] Naisbit RE, Rohr RP, Rossberg AG, Kehrli P, Bersier L-F 2012 Phylogeny versus body size as determinants of food web structure. Proc. R. Soc. B 279, 3291–3297. (10.1098/rspb.2012.0327) PMC3385719 22628467

[56] Watling JI, Donnelly MA 2006 Fragments as islands: a synthesis of faunal responses to habitat patchiness. Conserv. Biol. 20, 1016–1025. (10.1111/j.1523-1739.2006.00482.x) 16922218

[57] Wang Y, Ding P, Chen S, Zheng G 2013 Nestedness of bird assemblages on urban woodlots: implications for conservation. Landsc. Urban Plan. 111, 59–67. (10.1016/j.landurbplan.2012.11.008)

[58] Kadmon R 1995 Nested species subsets and geographic isolation: a case study. Ecology 76, 458–465. (10.2307/1941204)

[59] Triantis KA, Bhagwat SA 2011 Applied island biogeography. In *Conservation biogeography* (eds R Ladle, RJ Whittaker), pp. 190–223. Hoboken, NJ: Wiley & Sons.

[60] Stang M, Klinkhamer PG, Waser NM, Stang I, van der Meijden E 2009 Size-specific interaction patterns and size matching in a plant–pollinator interaction web. Ann. Bot. 103, 1459–1469. (10.1093/aob/mcp027) 19228701 PMC2701768

[61] Dehling DM, Töpfer T, Schaefer HM, Jordano P, Böhning-Gaese K, Schleuning M 2014 Functional relationships beyond species richness patterns: trait matching in plant–bird mutualisms across scales. Glob. Ecol. Biogeogr. 23, 1085–1093. (10.1111/geb.2014.23.issue-10)

[62] Thébault E, Fontaine C 2010 Stability of ecological communities and the architecture of mutualistic and trophic networks. Science 329, 853–856. (10.1126/science.1188321) 20705861

[63] R Core Team. 2014 R: a language and environment for statistical computing. R Foundation for Statistical Computing, Vienna, Austria. See http://www.R-project.org.

[64] Eddelbuettel D, François R, Allaire J, Chambers J, Bates D, Ushey K 2011 Rcpp: seamless R and C^++^ integration J. Stat. Softw. 40, 1–18.

[65] Eddelbuettel D, Sanderson C 2014 Rcpparmadillo: accelerating R with high-performance C^++^ linear algebra Comput. Stat. Data Anal. 71, 1054–1063. (10.1016/j.csda.2013.02.005)

