# Supplementary material for: Unmasking structural patterns in incidence matrices: an application to ecological data
Source: J R Soc Interface. 2019 Feb 6;16(151):20180747. doi: 10.1098/rsif.2018.0747 (PMC6408342; doi:10.1098/rsif.2018.0747)
Supplement: Supplementary Methods and Results [file rsif20180747supp1.pdf]

Unmasking structural patterns in incidence matrices: an application to ecological data.

Bramon Mora et. al.

Supplementary Methods

Null model overlap

An important aspect to consider when comparing the performance of multiple null models is how different the structure of the data generated by them is from the original data (Joppa et al., 2010). The reason is that there is generally a number of shared links between the empirical adjacency matrix and the generated data, and these shared links may be crucial to explain the observed non-random patterns (Joppa and Williams, 2011). In other words, the number of shared links approximates the amount of variation between the data generated by the null model and the original adjacency matrix, and therefore is indicative of the "effective degrees of freedom" of a particular randomization scheme. We define the degree of overlap between the structure of an adjacency matrix and a particular ensemble of randomized networks as the average number of empirical links conserved after the randomization process. That is, given the adjacency matrix A and a particular randomized matrix A^*, we estimate the number of shared links between them as

$$\Theta\left(A^*\right) = \sum_{ij} \left(A_{ij} - \frac{|A_{ij} - A^*_{ij}|}{2} \right). \qquad (1)$$

The typical overlap between an adjacency matrix and a random ensemble can be defined then as the mean proportion of links shared between the former and each of the randomized matrices.

Estimating food-web phylogenies

To quantify any evolutionary signal underlying food-web structure, we first needed to generate phylogenies for the different species. To do so, we started by taxonomically classifying all species according to NCBI database (http://www.ncbi.nlm.nih.gov/) by means of the *classification* function in the R package *taxize* (Chamberlain and Szöcs, 2013). Using this information, we could obtain the cladograms corresponding to the species' taxonomy using the *as.phylo.formula* function from *ape* (Paradis et al., 2004). While doing so, we considered all species with an indefinite taxonomic classification as outgroups (e.g., moss cells

and unidentified detritus). We then calibrated the resultant trees based on published data of the actual divergence time between species. Whenever possible, we dated the ancestral nodes of the cladograms—the most recent common ancestor of two given taxa—according to Hedges et al. (2006, 2015), which is a database of published molecular divergence times for a large number of species ($<$50000). Finally, the age of all remaining undated ancestral nodes was estimated according to the branch length adjustment algorithm *bladj* (Webb et al., 2008), which evenly sets the undated nodes between dated ones.

Generating structured food-web structures

To generate structured food-web data, we chose to use the niche model presented by Williams and Martinez 2000. Given a set of n species, the niche model simulates the structure of a food web by assigning a random 'niche value' from the interval $[0, 1]$ to all species that determines who is eaten by whom. In particular, every species i with niche value k_i is set to consume any species that have a niche value falling in a particular range r_i randomly centered between $r_i/2$ and k_i, where r_i is drawn from a beta distribution with $\alpha = 1$ and expected value $2c$, and c is the desired connectance for the food web.

Generating structured species assemblages

To generate nested species assemblages, we follow a twofold process. First, given a number of rows n, columns m and connectance c, we add interactions $m_ij = 1$ to generate a perfectly nested matrix M. In particular, we fill the matrix following Patterson and Atmar 1986 to obtain a perfectly nested structure. Second, we add noise to the interaction matrix. To do so, we go through every element m_{ij} of the matrix and switch it with a given probability p (i.e. we change every element $m_{ij} = 1$ to $m_{ij} = 0$ with probability p, and vice-versa)

Supplementary Results

Food webs and overlap

We evaluated the degree of overlap between the empirical food-webs and the data generated by the null models to ensure that the observed differences in the motif representation were not a consequence of the strengths of the imposed constrains (Rohr et al., 2014). That is, we wanted to verify that the aforementioned differences were not due to the number of shared links between the empirical and random structures but instead arose from the intrinsic properties of the adopted null hypotheses. We observed that the overlap between the empirical networks and the random ensemble representing the uninformed null model ranged from 39% to 65%. On the other side, the degree of overlap was consistently lower for the data generated by phylogenetically-informed null model, ranging from 53% to 73% when we used the estimated probabilities according to predator's diet and from 48% to 69% when we adopted the alternative prey's consumers perspective.

To verify that the these overlap differences were not the responsible for the different motif representations, we evaluated the trajectories followed by the z-scores when progressively randomizing a given food web. That is, we quantified the motif over- and under-representation according to random ensembles presenting different degrees of overlap. Using these trajectories, we studied the motif representation as a function of the average number of links shared by the empirical food webs and the data generated by the different null models. We observed that the motif composition of a particular food web was very robust to changes accounting for the species' phylogenetic relationships. On the other side, this empirical motif composition was very sensitive to the uninformed randomization, showing a very different pattern relative to the phylogenetically-informed one. Figure 1 shows an example of the pattern found for the motif describing exploitative competition in a particular network.

Finally, we also confirmed this result by artificially increasing the overlap shown by the data generated by the uninformed null model relative to the phylogenetically-informed versions. In order to ensure that the differences observed between the motif representations obtained using those null models were not due to their different degrees of overlap, we

artificially increased the overlap of the uninformed null model to match the overlap of the informed ones. To do so, we used a Markov chain Monte Carlo switching algorithm. Specifically, given an adjacency matrix A representing a food-web structure and a null hypothesis described by the ensemble of randomized networks $\langle A^* \rangle$, we progressively reallocated the links of each A^* according to A until obtaining a particular overlap between them. In this process, for example, two links $i \leftarrow j$ and $l \leftarrow m$ can become $i \leftarrow m$ and $l \leftarrow j$, provided that the overlap between A^* and A either increases or stays the same. Using this algorithm, we generated random ensembles describing the uninformed and the phylogenetically-informed null hypothesis with the same degree of overlap relative to the empirical food webs. Figure 2 shows an example of the results obtained when artificially increasing the overlap of the uninformed null model to match the overlap of the phylogenetically-informed null model based on the predator's diet.

Benchmark testing

The core idea behind benchmark testing null models is to compare their performance when looking at both structured and random data. Specifically, a null model is expected to show a structural pattern to be significantly represented in structured data (low type II error), and unsignificantly represented in random data (low type I error). The uninformed null model used in the main text follow a 'swap' algorithm (Connor and Simberloff, 1979), which has been largely used and tested effective in the past (Milo et al., 2003; Itzkovitz et al., 2004). Therefore, to test the performance of the correlation-informed null model, we studied how an informed null model and a misinformed null model (i.e. informed by a randomized correlation matrix) compared to the uninformed null model in structured and random data. We expected the misinformed null model to show similar results to the uninformed one. Ideally, we instead expected the informed null model to show similar results to the uninformed null model when analyzing random data but very different results when studying structured food webs. In particular, given the right correlation matrix, the informed null model should be able to reproduce the patterns found in structured data.

For the application of the correlation-informed null model on food webs, we first generated 1000 food webs using the algorithm defined in the Supplementary Methods section, and 1000

random matrices. We generated all these matrices randomly picking the number of species $n \in [0, 1]$ and connectance $c \in [0.05, 0.15]$. For each of the structured food webs, we used the niche values for all species to generate a correlation matrix. To do so, we assumed an exponential correlation structure and calculate the correlation matrix using the R package *nlme*. Then, we used an uninformed, a misinformed, a niche-informed null model to analyze the shape of both structured and random food webs (Fig. 3). Following the example in the main text, we studied the representation of all three-species food-web motifs, focusing on whether the null models showed the data to present significant or unsignificant patterns. We found the correlation-informed null model to perform almost exactly as the uninformed null model did (Fig. 3). We found the niche-informed null model, on the other side, to explain much better the motif pattern found in structured food webs while not producing any misleading results in randomized structures (low type I error; Fig. 3).

For the second application of the correlation-informed null model, we first generated 1000 species assemblages using the algorithm defined in the Supplementary Methods section, and 1000 random matrices. We generated all these matrices randomly picking the number of rows $n \in [20, 100]$, the number of columns $m \in [20, 100]$, the connectance $c \in [0.05, 0.15]$, and the added noise $p \in [0.1, 0.3]$ (see Supplementary Methods). Following the same procedure as in our previous example, we generate the correlation matrices assuming an exponential correlation structure, using the column order as our similarity measure in this case. Again, we used an uninformed, a misinformed, an informed null model to analyze the shape of both structured and random species assemblages (Fig. 3). Following the example in the main text, we studied the nestedness pattern observed in the simulated species assemblages. As in the test for the food webs, we found the misinformed null model to perform almost exactly as the uninformed null model did (Fig. 3), and the informed null model to better explain the nested pattern found in structured species assemblages while not producing any misleading results in randomized structures (low type I error; Fig. 3).

Supplementary References

S. A. Chamberlain and E. Szöcs. taxize: taxonomic search and retrieval in R. *F1000Research*, 2, 2013.

E. F. Connor and D. Simberloff. The assembly of species communities: chance or competition? *Ecology*, 60(6):1132–1140, 1979.

S. B. Hedges, J. Dudley, and S. Kumar. Timetree: a public knowledge-base of divergence times among organisms. *Bioinformatics*, 22(23):2971–2972, 2006.

S. B. Hedges, J. Marin, M. Suleski, M. Paymer, and S. Kumar. Tree of life reveals clock-like speciation and diversification. *Molecular Biology and Evolution*, 34(4):835–845, 2015.

S. Itzkovitz, R. Milo, N. Kashtan, M. Newman, and U. Alon. Reply to "comment on 'subgraphs in random networks' ". *Physical Review E*, 70(5):058102, 2004.

L. N. Joppa and R. Williams. The influence of single elements on nested community structure. *Methods in Ecology and Evolution*, 2(5):541–549, 2011.

L. N. Joppa, J. M. Montoya, S. Vicente, J. Sanderson, S. L. Pimm, et al. On nestedness in ecological networks. *Evolutionary Ecology Research.*, 12:35–46, 2010.

R. Milo, N. Kashtan, S. Itzkovitz, M. E. Newman, and U. Alon. On the uniform generation of random graphs with prescribed degree sequences. *arXiv preprint cond-mat/0312028*, 2003.

E. Paradis, J. Claude, and K. Strimmer. APE: analyses of phylogenetics and evolution in R language. *Bioinformatics*, 20(2):289–290, 2004.

B. D. Patterson and W. Atmar. Nested subsets and the structure of insular mammalian faunas and archipelagos. *Biological Journal of the Linnean Society*, 28(1-2):65–82, 1986.

R. P. Rohr, S. Saavedra, and J. Bascompte. On the structural stability of mutualistic systems. *Science*, 345(6195):1253497, 2014.

C. O. Webb, D. D. Ackerly, and S. W. Kembel. Phylocom: software for the analysis of phylogenetic community structure and trait evolution. *Bioinformatics*, 24(18):2098–2100, 2008.

R. J. Williams and N. D. Martinez. Simple rules yield complex food webs. *Nature*, 404 (6774):180, 2000.

Supplementary Figures

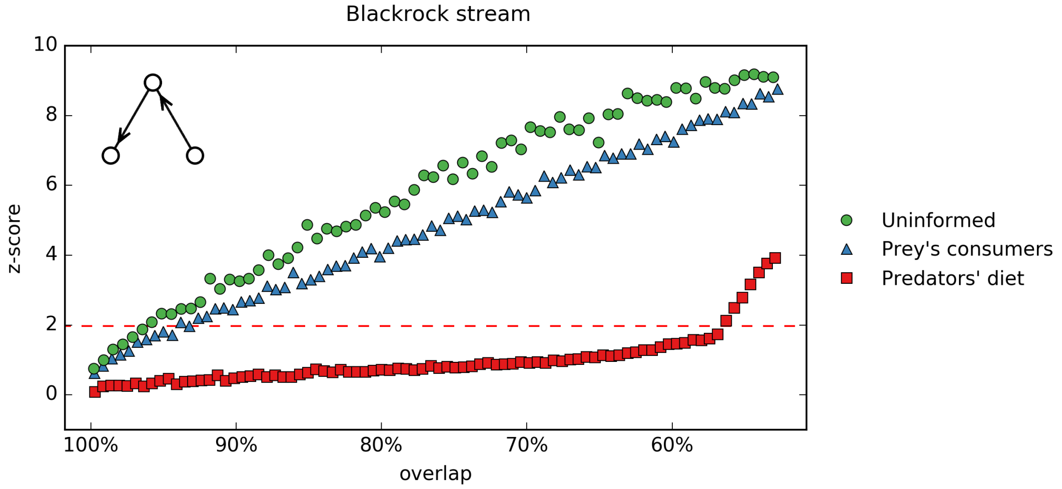

Supplementary Figure 1: The relationship between the motif representation of a simple food chain and the degree of overlap of the data generated by the null models for one of the empirical food webs studied here. The red and blue circles show the trajectories when the randomization accounts for the phylogenetic relationships in predators' diets and prey's consumers, respectively. Similarly, the green circles show the same according to the uninformed null model. The red dotted line indicates the threshold for significance $z = 1.96$.

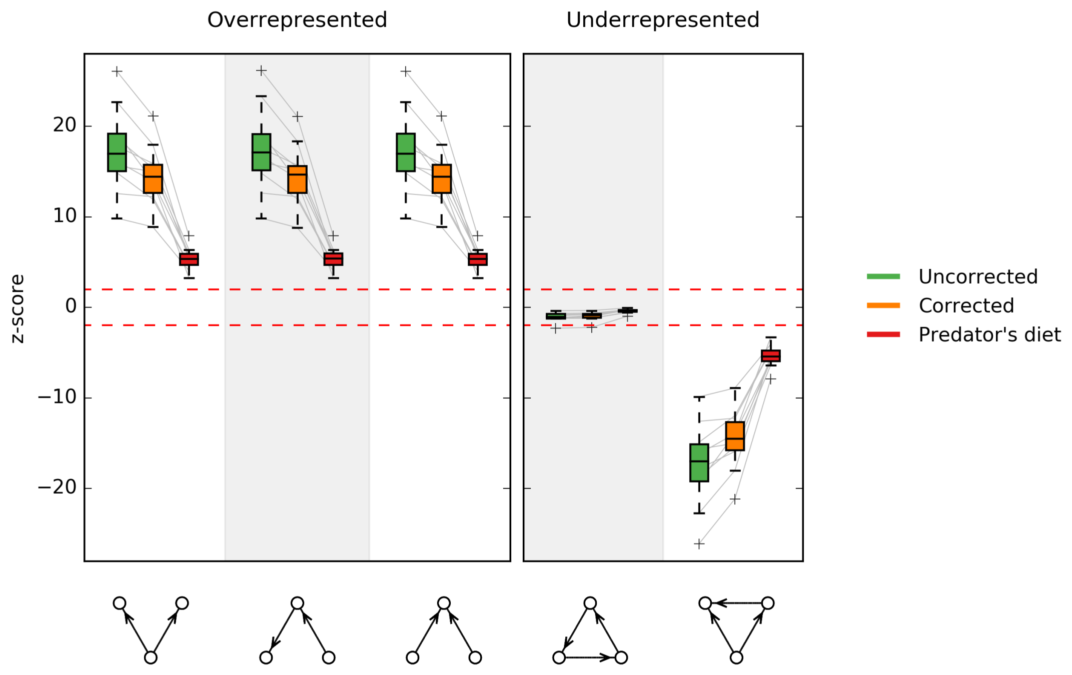

Supplementary Figure 2: The effect of the phylogenetic relationships between species on the motif representation of a set of food webs. For all motifs, the arrows indicates the transfer of energy from prey to predators. The red dotted line indicates the thresholds for significance $z \leq -1.96$ and $z \geq 1.96$. The boxes group all food webs, extending from the lower to upper quartile values of the data, with a line at the median (the grey lines connecting the boxes link the motif representation for the same food webs). The green boxes show the motif representation according to the uninformed null model when this has been not constrained. Similarly, the orange boxes show the same when the degree of overlap has been constrained. Finally, the red boxes contain the z-scores for each motif when the null model accounts for the phylogenetic relationships in predators' diets.

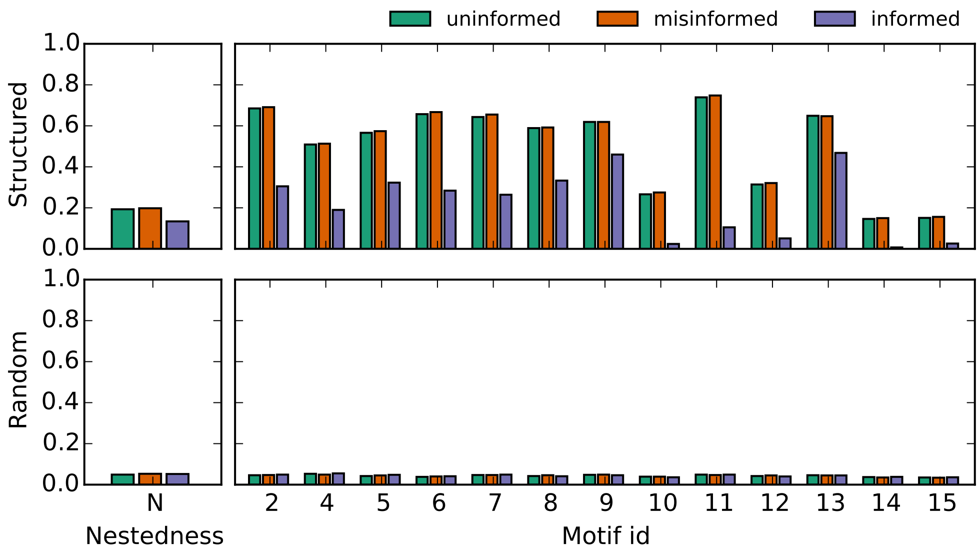

Supplementary Figure 3: Proportion of times the different null models showed a significant structural pattern in structured and random simulated data. For all cases, we studied the simulated data using three null models: an uninformed, a misinformed and an informed null model. Each bar represents the proportion of times the each model showed the data to present a non-random pattern. The two top panels show the results found for the study of nestedness and the motif representation in structured data, and the bottom panels show the same for random data. The different motif id characterize the distinct isomorphism classes defined by the function '`graph_from_isomorphism_class`' in the R package *igraph*.
